# “For Asia Market Only”: A Green Tattoo Ink between Safety and Regulations

**DOI:** 10.3390/molecules27113491

**Published:** 2022-05-29

**Authors:** Elvira M. Bauer, Daniele Cecchetti, Ettore Guerriero, Simone Quaranta, Francesca Ripanti, Paolo Postorino, Pietro Tagliatesta, Marilena Carbone

**Affiliations:** 1 Institute of Structure of Matter, Italian National Research Council (ISM-CNR), Via Salaria km 29.3, 00015 Monterotondo, Italy; elvira.bauer@mlib.ism.cnr.it; 2 Department of Chemical Science and Technologies, University of Rome Tor Vergata, Via della Ricerca Scientifica, 00133 Rome, Italy; daniele.cecchetti@uniroma2.it (D.C.); pietro.tagliatesta@uniroma2.it (P.T.); 3 Institute of Atmospheric Pollution Research, Italian National Research Council (IIA-CNR), Via Salaria km 29.3, 00015 Monterotondo, Italy; ettore.guerriero@iia.cnr.it; 4 Institute for the Study of Nanostructured Materials, Italian National Research Council (ISMN-CNR), Via Salaria km 29.3, 00015 Monterotondo, Italy; simone.quaranta@cnr.it; 5 Department of Physics, Sapienza University, P.le A. Moro 5, 00185 Rome, Italy; francesca.ripanti@uniroma1.it (F.R.); paolo.postorino@uniroma1.it (P.P.)

**Keywords:** green tattoo inks, composition, hydrophobicity/hydrophilicity, pigment load, phthalates, toxicity

## Abstract

Due to the increasing tattoo practicing in Eastern countries and general concern on tattoo ink composition and safety, the green tattoo inks Green Concentrate by Eternal, for European and “for Asia Market Only” were analyzed, under the premise that only the former falls under a composition regulation. A separation of the additives from the pigment was carried out by successive extraction in solvents of different polarities, i.e., water, acetone and dichloromethane. The solid residues were analyzed by IR and Raman spectroscopies, the liquid fractions by GC/mass spectrometry. The relative pigment load and element traces were also estimated. We found that the European and the Asian inks are based on the same pigment, PG7, restricted in Europe, though at different loads. They have a similar content of harmful impurities, such as Ni, As, Cd and Sb and both contain siloxanes, including harmful D4. Furthermore, they have different physical-chemical properties, the European ink being more hydrophilic, the Asian more hydrophobic. Additionally, the Asian ink contains harmful additives for the solubilization of hydrophobic matrices and by-products of the phthalocyanine synthesis. Teratogenic phthalates are present as well as chlorinated teratogenic and carcinogenic compounds usually associated to the laser treatment for removal purposes, to a larger extent in the European ink. The composition of the inks does not seem to reflect regulatory restrictions, where issued.

## 1. Introduction

The tattoo ink composition is becoming a growing matter of concern as a result of the increasing tattoo practice worldwide [1]. In fact, a growing number of studies is dedicated to their potential impact on several aspects such as health (especially allergies [2]), daily life issues such as the potential interference on MRI scans [3] and, ultimately, their removal [4]. Issues related on the potential toxicity of inks components are constantly raised [5].

Though tattoos became fashionable in western countries, eastern countries have recently been following the trend too [6,7].

Tattooing in Asian countries has a long history and variable perception in the populations depending on the period and on the country. For instance, in Japan in the 17th century (Edo period), it was a sign of distinction of Tobi (鳶, architect, security guards at festivals and firefighters) and Hikyaku (飛脚, delivery men). However, afterwards it started being used to mark criminals and, later on, to identify Yakuza members, thus assuming a completely different social meaning [8,9,10,11]. Also in imperial China, tattoos were used to blotch criminals (囚) [12], hence they were considered a social marking [13]. Nowadays, the practice of tattooing is rather varied. In countries such as South Korea, people sporting extensive tattoos are generally not allowed in public baths [14]. Nonetheless, the trend of tattooing practices is reported to be on the rise [15], and surveys on tattoos diffusion worldwide indicate a similar increase of tattoo practices in China [16]. Tattoos are also widely practiced in territories like Hong Kong, which had a strong western influence for a long time. Regulations on tattoo inks in Asian countries are relatively difficult to retrieve, mostly due to language barriers, and they may refer both to the way tattoos are practiced and on the inks’ compositions. Amidst language obstacles, we could ascertain, for instance, that in South Korea, officially, only licensed medical professionals are allowed to practice tattooing [17]. As for the tattoo ink compositions, we inquired the Standardization Administration of China (中国标准化管理总局) and the China National Accreditation Service for Conformity Assessment (中国合格评定国家认可委员会) on indication from the Italian–Chinese Chamber of Commerce (意中商会) and received indications from both institutions that there are currently no regulations in China [18]. Tattoo ink providers, such as Eternal Ink (based in Brighton, USA), have different distribution channels for Europe and Asia, the European ones being based in Munich, Germany; Sheffield, UK; and Amsterdam, The Netherlands, and the Asian ones in Hong Kong and Suzhou. In addition, bottles with different labels are provided. In particular, the ones for the Asian market are clearly marked as “FOR ASIA MARKET ONLY” and no indications are reported on the composition (Figure S1). At variance with this, bottles of tattoo ink for the European market usually report the composition, though not necessarily correctly [19], and are destined for countries where, in some cases, composition regulations are issued [20,21,22,23]. Due to the different labelling of tattoo ink bottles by the same producer for different countries, with different or no regulations, we purchased and analyzed the green ink Green Concentrate by Eternal Ink, Inc. in Italy through the hub in Munich, Germany and in Hong Kong, and compared the compositions. 

Tattoo inks are composed of a coloring agent, i.e., the pigment, and the vehicle to ensure the injectability of the pigment under the skin and provide components with biocide properties. In order to have a complete overview of the two Green Concentrate inks, the European (EGC) and the Asian one (AGC), we performed a procedure of the consecutive extractions of the inks and analyzed the pigment and the vehicle separately by IR, Raman and GC/mass spectrometry. Furthermore, an additional chromatographic column with chloroform separated a yellowish component from both inks, analyzed by semi-quantitative GC/mass spectrometry. Finally, the impurities content, such as metals or arsenic, was analyzed by XRF. This is a particular matter of concern, since their level in tattoo inks often exceeds the safety thresholds, where established [24,25,26]. 

Studies on tattoo ink composition typically focus on the detection of one of the components, i.e., they verify the pigment content, or, on a broader scale, they set up a method to determine the natures of the pigment(s) and create an associated database [27,28]. More often, metal residuals are analyzed [24,29,30,31,32,33,34] also because their concentration limits are clearly reported in the ResAP (2008) guidelines [35] and following amendments [36]. Volatile organic compounds, aldehydes [37] and phthalate content [26] in tattoo inks have been investigated too. In some cases, metal residual and pigments were determined in the same set of inks [30]. In the present paper, we analyze the whole composition of the selected inks, i.e., pigments, additives and trace elements. Furthermore, we focus on inks of a single producer for different markets, taking into account different legislation backgrounds. The aim is to discern possible market-driven differences in ink composition, especially in terms of toxic constituents. It must be added that, when dealing with tattoo inks, Asian countries are usually mentioned as production sites rather than as markets, and safety concerns are addressed regarding the production conditions and ingredients. Here we use a different approach and focus on different markets and a single producer.

By performing multiple analyses, we found that both inks contain PG7, which is restricted in some European countries, with an estimated 8% higher load in the Asian vs. the European ink. The analysis of impurities revealed the presence of toxic and allergenic elements, such as nickel, arsenic, cadmium and antimony, in comparable quantities in the two inks and exceeding the European permitted threshold. As for the additives, both inks contain cyclic siloxanes, including the teratogenic D4, a large variety of hydrocarbons and chlorinated compounds, some of which carry several types of hazards. However, only a few compounds are common to both inks. In general, the hydrophobic components are dominant in the Asian ink, hydrophilic ones are largely present in the European ink. Some, peculiar toxic compounds, such as dioxane, dioxolane and crown ethers as well as synthesis by-products as 4,5,6,7-tetrachloro-1,3-isobenzofuranedione are solely present in the Asian ink. Chlorinated harmful by-products, often associated to the phthalocyanine decomposition upon laser treatments are, actually, already present in the ink, by larger amount in the European than in the Asian one. Both inks contain different types of harmful phthalate, which impart an additional yellowish nuance. All in all, it is not safety regulations that drive the ink’s composition. It is possible, instead, that the market destination is determined by the selection of the inks based on physico-chemical properties, maybe connected to the pigment load.

## 2. Materials and Methods

The European Green Concentrate by Eternal Ink, Inc. was purchased at a local store in Rome, imported from the hub in Munich, Germany, the Asian Green Concentrate also by Eternal Ink, Inc. was purchased at a licensed tattoo ink provider in Hong Kong. The pigment PG7 (hexadecachloro copper phthalocyanine) was purchased from Kremer Pigmente GmbH, while Pigment PG36 (hexabromodecachloro copper phthalocyanine) was purchased from Schmincke Künstlerfarben GmbH (Erkrath, Germany). Dichloromethane, acetone, ultrapure water, chloroform, sulfuric acid and ethyl acetate were analytical grade, purchased by Merck KGaA (Darmstadt, Germany). 

Infrared spectra were recorded with a Shimadzu Prestige-21 FT-IR instrument, equipped with an attenuated total reflectance (ATR) diamond crystal (Specac Golden Gate), in the 400–4000 cm^−1^ range, with a resolution of 4 cm^−1^. Raman measurements were performed using a Horiba HR-Evolution micro-spectrometer in backscattering geometry, equipped with He-Ne laser, λ = 632.8 nm. The UV–Vis spectra were recorded with a Perkin Elmer, Lambda 950 spectrophotometer, using a quartz cuvette of 1 mm optical pathway. Gas chromatography–mass spectrometric analyses were performed using a triple quadrupole gas chromatograph/mass spectrometer (1310GC/TSQ 8000 Evo, Thermo Scientific, Waltham, MA, USA). The chromatographic separation was carried out with a DB/XLB column (60 m, 0.25 mm I.D., Agilent J&W, Santa Clara, CA, USA) with hydrogen as a carrier gas at a 3 mL min^−1^ flow rate. A 1 μL sample was injected in splitless mode at 250 °C. The oven program was the following: an isothermal at 90  °C for 5 min, followed by a ramp of 10  °C min^−1^ to 280  °C, which was maintained for 5 min. The mass spectrometer was operated on a 70 eV positive electron ionization (EI+) mode and at an emission current of 50 μA. The acquisition was performed in scan mode 35–450 m/z in 0.2 s. The transfer line and ion source temperatures were kept at 290  °C and 300  °C, respectively. GC/MS peak identification was conducted using the software Xcalibur 2.2 by Thermo Fisher Scientific.

Elemental chemical analysis of the powder samples was carried out by X-ray fluorescence (XRF) analysis by means of an energy dispersive X-ray fluorescence (ED-XRF) spectrometer (SPECTRO XEPOS HE XRF) optimized for heavy elements with 50 W Max power and Max 50 kV. The spectrometer was equipped with Pd/Co alloy tube. The results were obtained through the “TurboQuant powders and liquid” method (XRF Analyzer Pro software) and the SPECTRO procedure calibration model (a combination of the fundamental parameter and extended Compton scattering model with a calibration of the mass attenuation coefficient).

### Extraction Procedures

Two extraction procedures were adopted. In the first procedure, a quota of each ink was air dried, gently grinded in an agata mortar and subjected to an extraction in three subsequent steps with solvents of different polarities, i.e., water, acetone and dichloromethane. In more detail, the dried inks were suspended in distilled water, stirred for a few minutes and centrifuged. The procedure was repeated 3 times and the supernatant collected. The sediment was subsequently suspended in acetone, stirred overnight, centrifuged and the supernatant collected, before a new suspension was made in dichloromethane and the final stirring proceeded for 1 h, prior to the final centrifugation. Each fraction of supernatants was subjected the GC/mass analysis, provided that the water fraction underwent a further essential extraction with ethyl acetate, whereas the final sediments (labelled SedEGC for the European and SedAGC for the Asian inks) were analyzed by IR and Raman spectroscopy.

The second procedure was a chromatographic column in silica gel using chloroform as mobile phase. An amount of 6.6 g of silica gel (using the slurry method with petroleum ether) was used for the extraction of 484.4 mg of dried EGC and 438.6 mg of dried AGC samples with chloroform. A yellow component was separated in the elution column of both samples (Figure S2). The corresponding fraction was collected and subjected both to UV–Vis spectrometry and to GC/mass spectrometry, with the addition of 1 ng/mL of two deuterated internal standards, anthracene D10 and perylene D12. The semiquantitative assessment was performed using an average response factor, calculated over 17 chlorinated pesticides, i.e., α-BHC, γ-BHC, β-BHC, δ-BHC, aldrin, heptachlor, heptachlor epoxide isomer H, Endosulfan I (α), 4,4′-DDE, dieldrin, endrin ketone, 4,4′-DDD, endosulfan II (β), endrin aldehyde, 4,4′-DDT, endosulfan sulfate and metoxychlor, dissolved in chloroform and injected separately. All pesticides were purchased from Merck.

## 3. Results and Discussion

The composition of tattoo inks needs to be disclosed in some European countries, but such a requirement is not necessary either in the USA nor in Asian countries, (according to the information we could access). In order to gain insight into the composition and possible associated hazards, the tattoo inks were separated into their two main components, i.e., the pigment(s) and the vehicle, which were subsequently analyzed independently. The rationale behind the inks treatments is the extraction of all components of the vehicle, using solvents of different polarities and subsequent analysis both of the extracts and of the solid residues. A parallel chromatographic column in chloroform completed the set of extractions.

### 3.1. IR and Raman

**Figure 1:**
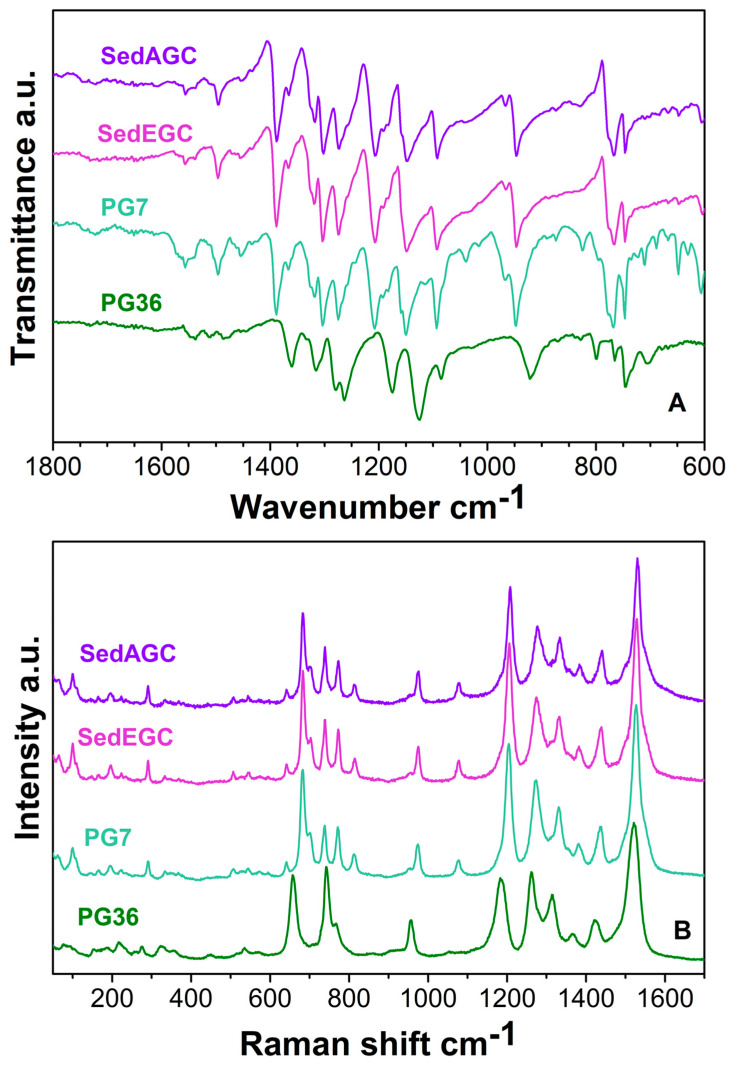
Analysis of the solid residue of Asian (SedAGC) and European (SedEGC) compared to the pigments PG7 and PG36. (**A**) IR spectra and (**B**) Raman spectra.

The solid residues SedEGC and SedAGC as well as the reference pigments PG7 and PG36 were analyzed by IR and Raman spectroscopy. The corresponding spectra are reported in Figure 1A,B. The choice of the reference pigments was made on the basis of the declared content on the bottle of EGC and what was actually found in the GC ink bottles [19,38].

In the IR spectra, almost all bands of PG7 in the 1000–400 cm^−1^ region were stronger when compared to PG36. The C-Cl vibrations fall in the 777–768 cm^−1^ range for PG7 and 775–765 cm^−1^ for PG36, whereas the broadening of the band around 745 cm^−1^ of the PG36 could be an effect of the C-Br bonds. Furthermore, PG7 have stronger bands at 606 cm^−1^. There was a one-to-one correspondence of the features of SedAGC, SedEGC and PG7, thus implying that both the European and the Asian ink contain the same pigment, i.e., PG7.

As far as Raman spectra are concerned, in the 1200–1600 cm^−1^ range, PG36 and PG7 have similar features, i.e., number, position within 15 cm^−1^ and intensity ratio of the peaks. The very strong peak that appears at 1186 cm^−1^ in the PG36 spectrum and is attributed to the isoindole in-plane bending [39] is shifted to 1203 cm^−1^ in PG7 spectrum. The largest differences between PG7 and PG36 occur in the 600–850 cm^−1^ range, where PG36 has a duplet at 662 cm^−1^ and 748 cm^−1^ and PG7 a quadruplet at 682 cm^−1^, 736 cm^−1^, 770 cm^−1^ and 812 cm^−1^, which could be considered fingerprints of the two pigments [40,41] and are attributed to macroring symmetric breathing, deformation and stretching. Additional features in the 100–600 cm^−1^ range also display some differences, but the features have, on average, low to very low intensity and the comparison in this region is less accurate.

The frequency shift observed in the PG36 and PG7 spectra are due to the different halogen atoms bound to the benzene rings; indeed, the substitution of some Cl atoms with more heavy Br ones produces a hardening of the indole vibrations towards lower wavenumbers.

Both SedAGC and SedEGC display a one-to-one correspondence to the PG7 Raman features, within 4 cm^−1^, a difference which can be related to instrumental error as well as to the contribution of residual additives [19]. Therefore, also Raman spectra indicate that both Asian and European inks contain PG7. 

The detailed assignments of the IR features can be retrieved from [19], whereas the assignment of the Raman features of PG7, PG36, SedAGC and SedEGC are reported in Table S1 of the Supplementary Information. 

**Figure 2:**
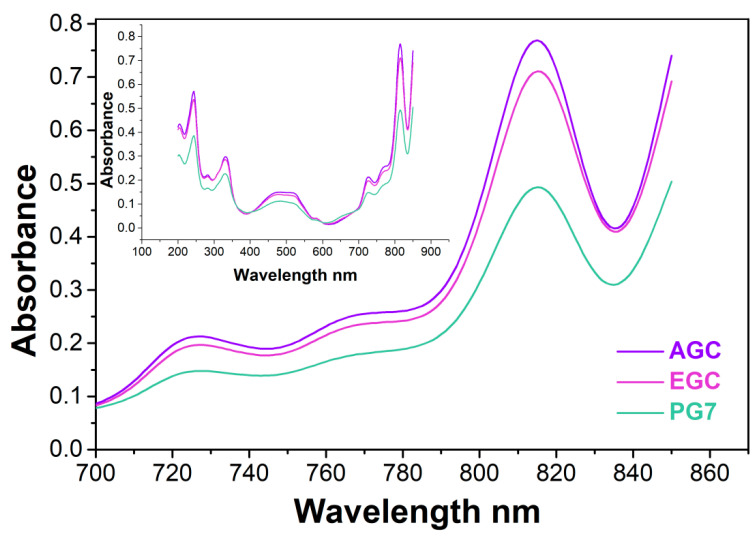
UV–Vis spectra of AGC, EGC and PG7 dissolved in concentrated sulfuric acid in the range 700–850 nm. The spectra in the whole 200–850 nm range are reported in the inset.

An estimate of the relative pigment content was obtained by UV–Vis spectroscopy. However, in this case the choice of solvent is crucial, due to different solubility and/or dispersibility of the various components of the inks in different solvents. Therefore, we opted for dissolving weighted amounts of each dried ink, PG7 and PG36, to reach a concentration of 0.09 mg/mL in concentrated sulfuric acid. This operation is associated with a significant bathochromic shift, due to the protonation on the outer four bridging nitrogen atoms which strongly polarizes the macrocycle, thus decreasing the energy of all electronic transitions [42,43]. As a consequence, the green ink dispersions turn into reddish to purple solutions (Figure S3). The UV–Vis spectra of the AGC and EGC solutions in sulfuric acid are reported in Figure 2 in an energy wavelength range 200–850 nm, along with PG7 for comparison purposes. 

AGC, EGC and PG7 all show absorptions at 283 nm, 331 nm, around 500 nm (broad), 579 nm, 675 nm (shoulder), 729 nm, 770 nm (shoulder) and 815 nm. These spectral features registered in sulfuric acid are identical to the ones observed by [44] although the most intense absorption band at 861 nm could not be observed in our case due to the detector limits of the spectrophotometer used in our study. An assessment of the relative pigment content was carried out through the comparison of the highest feature of the spectra at 815 nm, which is 8% more intense in AGC than in EGC (the same intensity ratio is kept throughout the whole spectra, but it is more accurately measured at the peak). PG7, which is sometimes referred to as a “pure” pigment, is 50% less intense than AGC, thus indicating that it does contain additives in larger amounts in comparison to the inks.

### 3.2. Elemental Content by XRF

The residual content of harmful elements in tattoos and permanent makeup is also a matter of concern and the ResAP (2008) provides indications on their threshold levels, i.e., the maximum allowed concentrations of impurities, based on several parameters, including relevant toxicological studies on cosmetic ingredients, acute toxicity and carcinogenicity. The elemental content in EGC and AGC was estimated by XRF and the results are reported in Table 1 for the elements whose limits are provided in the ResAP(2008) guidelines [35] and amendments [36] (also reported in Table 1).

A complementary list of analysed elements not subjected to ResAP regulations is reported in Table S2. In addition, the element content in tattoo inks from previous investigations is reported in Table 1 for comparison purposes.

Two major aspects emerge from Table 1: the concentrations of nickel, arsenic and antimony are well above the limit, the concentration of cadmium is on the edge and there are traces of chromium, though XRF cannot distinguish between Cr(VI) (more harmful) and Cr(III) (less harmful). The concentrations of residual elements are substantially similar in the two inks. The hazards associated with these elements have been long known and documented [45,46,47,48], and 2020 ResAP amendment requires that the tattoo ink labels carry the text “contain chromium and nickel, they can cause allergic reactions”, even if their content is lower than the limit imposed. It must be added that copper also largely exceeds the European limit, though the threshold is higher for many other elements [45]. The copper concentration in the inks is inevitably high since it is a constituent of the pigment. Furthermore, it is complexed by the phthalocyanine, whereas the maximum allowed level refers to the soluble copper in the ink preparations and XRF analysis fails to distinguish between the two. The presence of other elements, such as aluminum and silicon, is likely related to the addition of dispersants in the ink formulations and is also slightly higher in the European than in the Asian ink. In Table 1, the element traces in tattoo inks analyzed in previous investigations are also reported. In these papers, either inks produced worldwide (Japan, the USA and China [30], for instance) or purchased in various geographic areas (Baghdad [32], Iran [31] and Korea [26]) were selected. The values reported in Table 1 refer to green inks, if this information could be extracted. In all other cases, the average value over all inks is reported. Finally, the paper by Wang et al. [30] analyzed 73 different inks. In this case, the frequency of inks exceeding the ResAP limits is reported. In general, it appears that As, Cd, Sb and Ni tend to be above the EU limit also in other analyzed inks, indicating a diffuse problem of production and routine check of tattoo inks. 

### 3.3. GC-Mass Spectrometry

The gas–chromatography analyses of the sequential extractions reveal the presence of hundreds of components. A selection of compounds is reported in Table 2, with hazards related to carcinogenicity, organ and fertility damage, i.e., H340, H350, H351, H360, H361, H370, H372 and H373. The codes’ correspondence is reported in Table S3. A color code is used to identify the toxicity. Most notably, red labels in a black field indicate carcinogenicity; red labels in a green field, damage to organs; bluish labels in purple field, allergic reaction to the skin; and bluish ones in black field, fatal in contact with skin.

The complete color code is reported in the Supplementary Information. For some compounds no hazard information was available and we marked them as NA (not available). Additionally, the Sigma SDS were investigated and if the compound was generally reported as non-dangerous, it was labelled as NDAS (non-dangerous according to Sigma). The whole list of compounds is reported in the Supplementary Information, in Tables S4–S6, for water, acetone and dichloromethane extracts, respectively. A few compounds were extracted in both acetone and chloroform and are labelled white in a dark red field in the tables. The analysis of all the extracts indicates the presence of siloxanes D3 through D9 in both inks. Siloxanes are dispersing agents usually employed for pigments in paintry and tattoo inks [28]. They can be both linear (usually labelled with an “L” followed by a number) and cyclic (labelled with a “D” followed by a number), the latter being more toxic, in particular the D4, teratogenic. Furthermore, siloxanes may resist laser treatments for removal purposes [19,49]. As far as hydrocarbons are concerned, the overall analysis indicates a difference in the hydrophilic/hydrophobic components’ ratio between the two inks, with a larger number of water extract components and hydrophilic components in EGC as compared to AGC. EGC contains a large number of different alkenes, alcohols and long-chain carboxylic acids, whereas AGC has a larger number of different alkanes. As for the other components, AGC has more ketones (mostly extracted in acetone) and nitroderivatives. The higher hydrophobicity of AGC is coupled to the presence of dioxane and dioxolane, which are suited for the dispersion and solubilization of dyes, polymeric and hydrophobic compounds [50] but are also toxic. Dioxane is used as stabilizer for chlorinated solvents, such as 1,1,1-trichloroethane. It is also used as solvent for dyes, paints, resins, varnishes and waxes [51] but it was banned in cosmetics due to carcinogenicity in animals [52,53] and, consequently, suspected carcinogenicity in humans. Dioxolane is also a typical solvent for softening and dissolving polymers and is used for improved dye retention by ester fibers [28]; it has been classified as potentially damaging for fertility and unborn children (H360). Pentachloroaniline, a carcinogenic (H373), is present in both inks. Naphthalene, a PAH known for its carcinogenicity, is present in EGC, along with 1-methylnaphthalene (H373, organ damaging), whereas the 2,6-diisopropylnaphthalene is “only” harmful if swallowed (H302) and is also present in both inks. Benzene derivatives of different types are present in the two inks. Branched derivatives, such as 1-(1-methylethenyl)-4-(1-methylethyl)-benzene, are prevalent in the Asian ink.

Compounds detected only in the Asian ink are 15-Crown-5 and 12-Crown-4, the latter fatal if inhaled, 4,5,6,7-tetrachloro-1,3-isobenzofuranedione. The employment of crown ethers in tattoo inks is rather unique. When derivatized, crown ethers can be used in association with dyes in inkjet printing fluids [54] as a hook to the paper. 

4,5,6,7-tetrachloro-1,3-isobenzofuranedione is a fully chlorinated phthalic anhydride, carcinogenic, teratogenic and causes allergic reactions of the skin. Most likely it is a by-product of the chlorination of residual reagents of the phthalocyanine synthesis [55], thus implying a bypass of the purification step, prior to the chlorination procedure. 

The type of enrichment of hydrophilic or hydrophobic compounds in an ink, eventually also plays a role in view of a possible removal. Recently a comparative study was performed of EGC ink treatment with five different type of lasers, i.e., a Ruby nanosecond laser and Nd:YAG nano- and picosecond lasers, both in normal and array mode, irradiating the same total energy. It was discovered that the hydrophilic component of the ink, such as the alcohol molecules, emerged almost exclusively in case of the Nd:YAG nanosecond treatments, with a preference for linear alcohols in normal mode operation and branched alcohols in array mode operation [49].

### 3.4. Analysis of the Extracts

**Figure 3:**
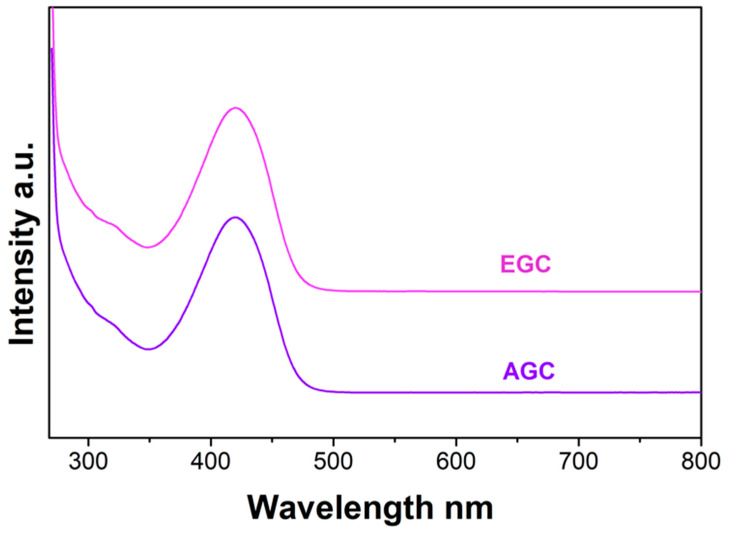
UV–Vis spectra of the yellow fraction of the Asian (**AGC**) and European (**EGC**) inks eluted with chloroform. The spectra have been vertically shifted for a better outlook.

The composition analysis was completed by column chromatography in chloroform, which revealed the presence of a yellow component in both inks (Figure S2). The UV–Vis spectra of the eluted fractions are reported in Figure 3 and are characterized by a peak at 420 nm in both cases. This component is likely hidden in the UV–Vis spectra of the inks by the shoulder of the Soret band of the chlorinated copper phthalocyanines, which appears at 410 nm for inks dispersed in DMSO [56].

The compounds eluted by chromatographic column in chloroform are reported in Table 3, along with the concentration ratio between the European and Asian ink where it could be assessed. It must be added that, with the determination method we used, we could only estimate the compound concentrations. The concentration ratio between the inks is, however, reliable. The semiquantitative concentration of the eluted compounds is reported in Table S7. 

The chromatographic column also reveals the presence of compounds which were not evidenced by the successive extraction procedure. In particular, a large number of chlorinated compounds is present, nearly all with hazard codes indicating carcinogenicity and/or teratogenicity. In more detail, tetrachloroethene, pentachloroethane, hexachloroethane, terbuthylazine, pentachlorophenol and hexachlorobenzene are present in both inks.

The ratio of these compounds between the two inks ranges from between 0.13 to 14.86 for hexachlorobenzene and pentachloroethane, respectively. The semiquantitative analysis of pentachloroaniline indicates a larger presence in the Asian ink. Among the chlorinated compounds, 2,4-dichloro-1,1-biphenyl (carcinogenic, H373) was detected only in the Asian ink. It must be noted that compounds such as hexachlorobenzene (carcinogenic and teratogenic) have often been associated with the decomposition of PG7 upon laser treatments [38] or with the interaction between the pigment and hydrocarbons in pyrolysis and laser treatments [56]. Though this might also be the case, the compound is definitely already present in the inks before any treatment, thus adding to the dangers associated with tattoos.

The yellowish color of the eluted compound cannot be reconducted to routinely used chlorinated yellow pigments such as PY14 or PY138. Of all compounds, phthalates are the most likely candidates to impart a yellowish color to the chloroform solution. We were able to single out different phthalates, two of which are common to both inks, the butyl 2-pentyl phthalate and the diethyl phthalate, both of which do not report hazard indications (codes not available). Diisooctyl phthalate is present in the EGC and dibutyl phthalate in the AGC, both are suspected of being damaging to unborn children (H361). However, the combined exposure to multiple phthalates is deemed to be underestimated [57]. It cannot be ascertained whether phthalates are a left-over of the phthalocyanine synthesis, from storage in plastic bottles or if they are intended as a color correction upon ageing. However, the presence of phthalates in tattoo inks is an open issue, since their presence was also ascertained in a set of Korean inks [26].

## 4. Conclusions

A comparative analysis has been made of the ink Green Concentrate by Eternal “for Asia Market only” and for the European market. The overall analysis indicates that the two inks have the same pigment, though at different loads, similar levels of impurities and the presence of siloxanes, whereas differences appear in the hydrocarbon composition. The major difference of the hydrocarbon components is related to the overall hydrophilic/hydrophobic character. The Asian ink, which is more hydrophobic, also contains aprotic (harmful) solvents for the solubilization of polymers and dyes and a larger content of pigment. A hypothesis compatible with the findings is that the two inks have a single or similar formulation at start, which is followed by a selection of the hydrophilic “lighter colored” fractions for the European market and the hydrophobic “darker colored” fractions for the Asian market. Carcinogenic phthalates and chlorinated compounds or by-products are present in both inks. Some of them, such as pentachloroethane, are more present in the European ink; others, such as hexachlorobenzene, are more present in the Asian ink. Left-overs of the phalocyanine synthesis and chlorination, such as 4,5,6,7-tetrachloro-1,3-isobenzofuranedione, were found in the Asian ink only. Different harmful phthalates were found in the two inks, which contributed to an additional yellow nuance. The European market, or at least a part of it, has tattoo ink composition regulations, at variance with what information we could gather regarding the Asian market. However, the composition of the inks does not seem to reflect regulatory restrictions, where issued. Asian and European Green Concentrate by Eternal contain different, equally harmful components. Since the presence of harmful components in tattoo inks is a matter of concern, it would be advisable to define the minimum requirements of marketed tattoo inks and perform routine tests using validated methods. It must be added that the toxicity of the various components upon injection and residence under the skin is not always known. Therefore, it can be foreseen that future developments will include aspects related to the toxicological effects of tattoo inks components, in vitro and in vivo.

## Figures and Tables

**Table 1:** Total concentration (μg/g) of various elements in EGC, AGC and reference papers, along with the limits recommended in the European Union. The elements marked in bold exceed or are very close to the limit indicated in the ResAP2008 and amendments. The star * indicates that data for the green ink were extracted from the reference paper. ND = not detected, DL = detection limit, RC = restricted concentrations; OA = element occasionally above the limit; AF = element present in a few inks; AA = element almost always present.

Metal	EGC	AGC	[29] *	[24]	[26]	[32] *	[31] *	[30]	EU limits *
Chromium	<0.1	<0.1	0.22	<DL	6.1 ± 7.7		170	AA	0.5
Cobalt	<0.1	<0.1	ND		1.7 ± 4.6			OA	0.5
**Nickel**	**12.0**	**11.7**	0.14	<DL	5 ± 8.7		6.8	3/59	**5**
Copper	38610	33440	3882	4400 ± 200	1840 ± 5040		63	1	250
Zinc	9.9	<0.1	0.98		8.7 ± 23.6	5.23		<RC	2000
**Arsenic**	**2.4**	**2.3**	ND		2.7 ± 6			OA	**0.5**
Selenium	<0.1	<0.1	1.66						2
**Cadmium**	**0.5**	**0.7**	0.06	<DL	0.6 ± 1.9	0.83	1.617	<RC	**0.5**
**Antimony**	**1.9**	**2.6**	ND		1.6 ± 4.5			<RC	**0.5**
Barium	19.3	12.2	18.1		9.8 ± 18.8				500
Mercury	<0.2	<0.2	0.06	<DL	0.0027 ± 0.0034			AF	0.5
Lead	<0.8	<0.8	0.17	0.80 ± 0.04	1.6 ± 5.2	6.3	2.27	AF	0.7

**Table 2:** Selection of the most hazardous compounds extracted in water, acetone and CCl_2_H_2_, and corresponding hazard codes. Chlorinated compounds are reported in **violet**, phthalates in **orange** and peculiar compounds of AGC in **light blue**. The colours assigned to different hazards indicate: **Red = lethality**, **Orange = Toxicity**, **Pink = Harmfulness**, **Bluish = skin related issues**, **Green = irritation, damage, drowsiness**.

RT min	Compound	Hazards	EGC	AGC
	**Extraction in H_2_0**			
**3.87**	Octamethylcyclotetrasiloxane (D4)	**H361f(2)**	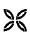	
**4.90**	2-methyl-1-propanol	**H302(4) H332(4) H350(1B)**		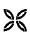
	**Extraction in Acetone**			
**2.09**	**1,4-dioxane**	**H319(2) H335(3)** **H351(2)**		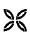
**3.37**	Styrene	**H315(2) H319 (2) H332(4) H372(1) H361d (2)**	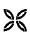	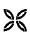
**3.83**	Octamethylcyclotetrasiloxane (D4)	**H361f (2)**	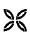	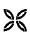
**8.40**	3,3,5-trimethyl-2-cyclohexen-1-one	**H302(4) H3 12 (4) H319(2) H335(3)** **H351(2)**		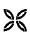
**9.56**	Naphthalene	**H302(4) H351 (2)**	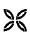	
**11.67**	1-methylnaphthalene	**H302(4) H319(2) H335(3) H336/ H373(2)**	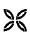	
**11.80**	**1,3-dioxolane**	**H319(2) H360(1B)**		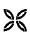
**19.17**	**Pentachloro aniline**	**H301(3) H3 11 ( 3) H331(3) ** **373(2)**	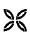	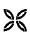
**19.27**	**12-crown-4**	**H330(1)**		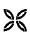
**21.27**	**4,5,6,7-tetrachloro-1,3-isobenzofuranedione**	**H317(1) H318(1) H334(1) H350(1A) ** **H373(2)**		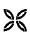
	**Extraction in CH_2_Cl_2_**			
**3.90**	Octamethylcyclotetrasiloxane (D4)	**H361f(2)**	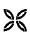	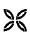
**18.65**	**Dibutyl phthalate**	**H360 DF (1B)**		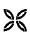
**19.22**	**Pentachloro aniline**	**H301(3) H3 11 ( 3) H331(3) ** **H373(2)**	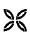	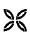
**24.87**	**Diisoocyl ** **phthalate**	**H360 DF (1B)**	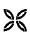	

**Table 3:** Compounds eluted from the chromatographic column with chloroform, corresponding hazard codes and concentration ratio between AGC and AGC. Chlorinated compounds are reported in **violet**, phthalates in **orange** and peculiar compounds of AGC in **light blue**. NA = not available, NDAS = non-dangerous according to Sigma. The star * indicates that data for the green ink were extracted from the reference paper. The colours assigned to different hazards indicate: **Red = lethality, Orange = Toxicity**, **Pink = Harmfulness**, **Bluish = skin related issues**, **Green = irritation, damage, drowsiness**.

RT Min	Compound	Hazards	EGC	AGC	EGC/AGC Ratio
**3.07**	**Tetrachloroethene**	**H315(2) ** **H319(2) H317(1)** **H336(3) H351(2)**	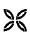	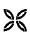	
**3.55**	**1,1,2,2-tetrachloroethane**	**H330(2) H310(1)**	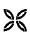	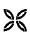	
**4.14**	**Pentachloroethane**	**H351(2) H372(1)**	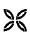	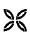	14.86
**5.88**	**Hexachloroethane**	**H319(2) H351(2)**	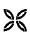	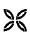	0.93
**8.29**	4-ethyl benzaldehyde	NDAS	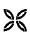		
**9.01**	1-isocyanato-2-methoxy benzene	NA	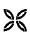		
**9.06**	1,3-di-tert-butyl benzene	NDAS		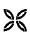	
**9.29**	**Terbuthylazine**	**H302(4) ** **H373(2)**	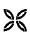	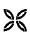	
**9.43**	1,4-dichloro-2-ethenyl benzene	NA	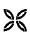	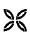	
**10.32**	**3,4,6-trichloro-2-methyl phenol**	NA	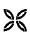	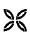	1.55
**10.33**	1-(dichloromethyl)-3-methyl benzene *	NA	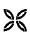		
**10.57**	**2-chloro-4-(chloromethyl)-1-methylbenzene**	NA	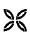		
**10.70**	**1,2-dichloro-4-(1-chloroethyl) benzene**	NA	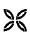	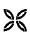	
**11.86**	Tetradecamethyl cycloheptasiloxane (D7)	**H319(2)**	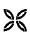	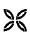	
**11.93**	**1,3,5-trichloro-2,4,6-trimethyl benzene**	**H315(2) ** **H319(2) H335(3)**	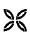	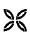	
**12.40**	**3,4-dichlorophenyl thiocyanate**	NA	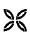	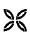	
**12.87**	2,6-di-*t*-butyl-1,4-benzoquinone	**H315(2) ** **H319(2) H335(3)**		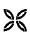	
**13.19**	**3-chlorobenzamide**	**H302(4) H312(4) H315(2) ** **H319(2) H332(4) H335(3)**	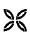		
**13.21**	2,5-di-*t*-butylphenol	**H315(2)** **H319(2) H335(3)**		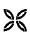	
**13.44**	3,3-dimethyl-1-(3H)-isobenzofuranone	**H315(2)** **H319(2) H335(3)**	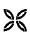		
**13.46**	Hexamethyl benzene	NDAS		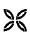	
**13.93**	Hexadecamethyl cyclooctasiloxane (D8)	**H319(1)**	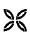	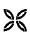	
**14.03**	**Pentachlorobenzene**	**H302(4)**	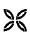	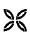	0.57
**14.04**	**3,4-dichloro benzamine**	**H301(3) H311(3) H317(1)** **H318(1) H331(3)**	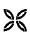		
**14.65**	**Diethyl phthalate**	NDAS	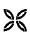	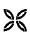	2.33
**15.81**	2,3-diphenyl-2-butene	NA		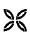	
**15.99**	**2,4-dichloro-1,1′-biphenyl**	**H373(2)**		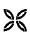	
**16.48**	**Hexachlorobenzene**	**H350(1B) H372(1)**	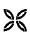	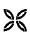	0.13
**16.78**	**2,6-dibromo-4-chloroaniline**	**H315(2) ** **H319(2) H335(3)**		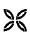	
**17.04**	1,4-dimethyl anthracene	NA		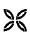	
**17.53**	**Anthracene D10**	**Internal standard**			
**17.63**	**Butyl tridecyl phthalate**	NA	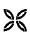	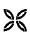	
**17.65**	**Pentachlorobenzonitrile**	**H315(2)** **H319(2) H335(3)**	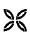	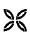	
**17.80**	1,1-(4,4′-diethyl)diphenylethane	NA	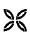		
**18.01**	**4,5-dichloro phthalimide**	**H315(2)** **H319(2) H335(3)**	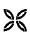		
**18.11**	**Pentachloroaniline**	**H301(3) H311(3)** **H331(3) H373(2)**	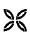	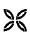	0.12
**18.24**	7,9-Di-tert-butyl-1-oxaspiro[4,5]deca-6,9-diene-2,8-dione	**H315(2) ** **H319(2A) H335(3)**	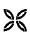	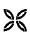	0.56
**18.28**	**Trichlorobenzenamide**	NA	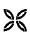	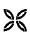	6.00
**18.63**	**2,3,4,5-tetrachloro aniline**	**H302(4) H315(2) H317(1) H318(1) H335(3)**		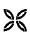	
**18.68**	**Butyl 2-pentyl phthalate**	NA	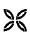	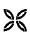	1.60
**18.71**	**2,3,4,5,6-pentachloro-N-(dichloromethylene)-benzenamine**	NA		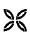	
**18.96**	**Pentachlorophenol**	**H301(3) H311(3) H315(2)** **H319(2)H330(2) H335(3) H351(2)**	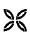	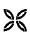	0.77
**19.93**	**Tetrachlorobenzamide**	NA	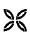	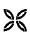	1.46
**22.04**	**Pentachlorobenzamide**	**H302(4) H312(4) H315(2) ** **H319(2) H332(4) H335(3)**	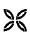	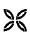	1.05
**22.61**	**3,4,5,6-tetrachloro phthalimide**	**H315(2)** **H319(2) H335(3)**	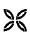	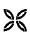	0.46
**23.79**	**Diisoctyl phthalate**	**H360(1B)**	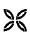	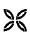	
**27.75**	**Perylene D12**	**Internal standard**			

## Data Availability

Not applicable.

## Supplementary Materials

The following supporting information can be downloaded at: https://www.mdpi.com/article/10.3390/molecules27113491/s1, Figure S1: Green Concentrate bottle from the Eternal Ink & Inc. purchased in Hong Kong. The text on the side of the bottle reports indications on how to perform an allergy test, prior to the injection of the ink under the skin. No composition of the ink is reported; Figure S2: Yellow fraction of the chromatographic columns of AGC and EGC eluted with chloroform; Figure S3: PG36, PG7, AGC and EGC solutions in sulfuric acid, in concentrations 0.09 mg/mL; Table S1: Raman shifts of PG7 and PG36 and corresponding assignments; Table S2: Mass percentages of elements analyzed by XRF in EGC and AGC and not subjected to RESAP recommended limitations; Table S3: Correspondence between hazard codes and toxicity; Table S4: Compounds extracted in water and corresponding hazard codes; Table S5: Compounds extracted in acetone and corresponding hazard codes; Table S6: Compounds extracted in CCl_2_H_2_ and corresponding hazard codes; Table S7: Compounds eluted from the chromatographic column with chloroform, and concentrations estimated with a semiquantitative method.
