# Supplementary material for: “For Asia Market Only”: A Green Tattoo Ink between Safety and Regulations
Source: Molecules. 2022 May 29;27(11):3491. doi: 10.3390/molecules27113491 (PMC9182426; doi:10.3390/molecules27113491)
Supplement: Supplementary file 1 [file molecules-27-03491-s001.zip › molecules-1716182-supplementary.pdf]

Supplementary Information

"For Asia Market Only": A Green Tattoo Ink between Safety and Regulations

Elvira M. Bauer [1,†], Daniele Cecchetti [2,†], Ettore Guerriero [3], Simone Quaranta [4], Francesca Ripanti [5], Paolo Postorino [5], Pietro Tagliatesta [2] and Marilena Carbone [2,*]

[1] Institute of Structure of Matter, Italian National Research Council (ISM-CNR), Via Salaria km 29.3, 00015 Monterotondo, RM, Italy; elvira.bauer@mlib.ism.cnr.it
[2] Department of Chemical Science and Technologies, University of Rome Tor Vergata, Via della Ricerca Scientifica, 00133 Rome, Italy; daniele.cecchetti@uniroma2.it (D.C.); pietro.tagliatesta@uniroma2.it (P.T.)
[3] Institute of Atmospheric Pollution Research, Italian National Research Council (IIA-CNR), Via Salaria km 29.3, 00015 Monterotondo, RM, Italy; ettore.guerriero@iia.cnr.it
[4] Institute for the Study of Nanostructured Materials, Italian National Research Council (ISMN-CNR), Via Salaria km 29.3, 00015 Monterotondo, RM, Italy; simone.quaranta@cnr.it
[5] Department of Physics, Sapienza University, P.le A. Moro 5, 00185 Rome, Italy; francesca.ripanti@uniroma1.it (F.R.); paolo.postorino@uniroma1.it (P.P.)
* Correspondence: carbone@uniroma2.it; Tel.:+39-06-7259-4470
† These authors contributed equally to this work.

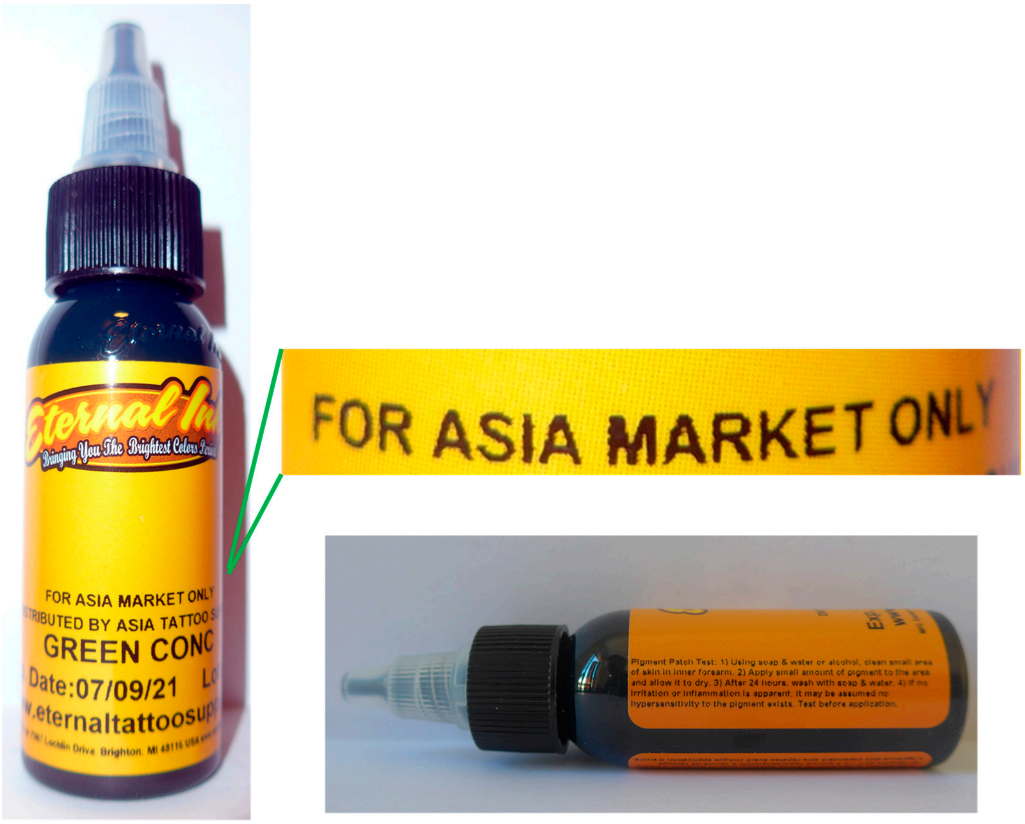

Figure S1 Green Concentrate bottle from the Eternal Ink & Inc. purchased in Hong Kong. The text on the side of the bottle reports indications on how to perform an allergy test, prior to the injection of the ink under the skin. No composition of the ink is reported.

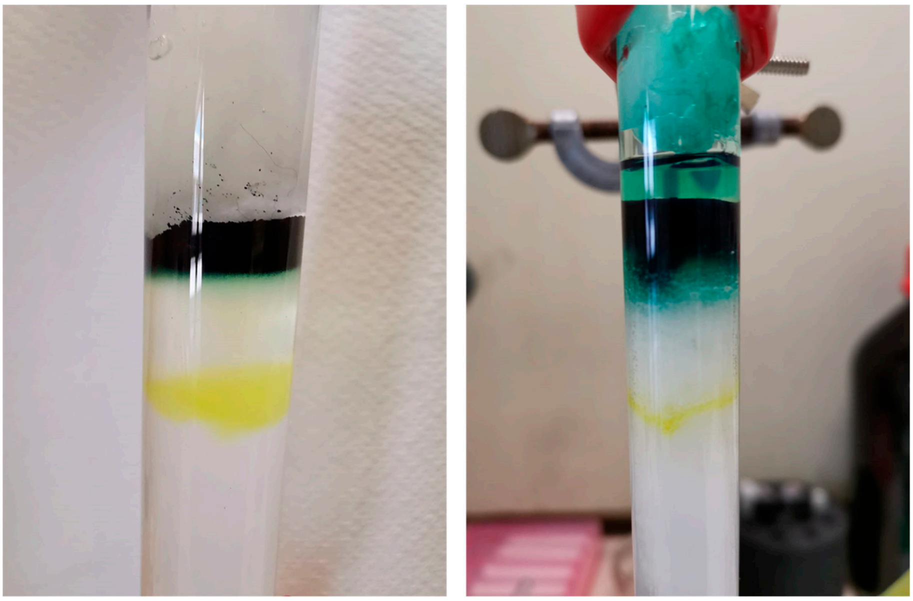

Figure S2 Yellow fraction of the chromatographic columns of AGC and EGC eluted with chloroform.

Table S1 Raman shifts of PG7 and PG36 and corresponding assignments. The intensities are indicated as: vw = very weak, w = weak, m = medium, s = strong, vs = very strong, sh = shoulder.

**PG36**	PG7	**SedAGC**	SedEGC	**Assignment**
**Raman shift (cm^{-1})**	Raman shift (cm^{-1})	**Raman shift (cm^{-1})**	Raman shift (cm^{-1})	
	100 (m)	**100 (m)**	100 (m)	Cu-N stretching
	145 (vw)	**146 (vw)**	148 (vw)	Cu-N stretching
**155 (vw)**				Cu-N stretching
	166 (vw)	**165 (vw)**	166 (vw)	isoindole in-plane stretching
**187 (vw)**	196 (vw)	**196 (vw)**	197 (vw)	isoindole in-plane stretching
**219 (w)**	223 (vw)	**223 (vw)**	223 (vw)	isoindole ring deformation
	235 (vw)	**236 (vw)**	234 (vw)	Cu-N stretching & pyrrole rotation
**258 (vw)**				Cu-N stretching & isoindole breathing
**276 (vw)**	291 (w)	**291 (w)**	291 (w)	Cu-N stretching
**322 (w)**	349 (vw)		347 (vw)	Cu-N stretching
**356 (vw)**	367 (vw)		368 (vw)	Cu-N stretching
**452 (w)**				isoindole ring deformation &

				C-C bending
	507 (vw)	507 (vw)	506 (vw)	isoindole ring deformation
	528 (vw)	529 (vw)	527 (vw)	isoindole ring deformation
534 (vw)	544 (vw)	544 (vw)	544 (vw)	isoindole ring deformation
571 (vw)	573 (vw)	573 (vw)	573 (vw)	C-C macroring breathing
	596 (vw)	597 (vw)	596 (vw)	C-N-C macroring breathing
	641 (m)	641 (m)	641 (m)	C-C macroring stretching & pyrrole out-of-plane stretching
662 (vs)	682 (vs)	683 (vs)	683 (vs)	macroring symmetric breathing
	698 (m)	701 (m)	703 (m)	macroring symmetric breathing
	736 (m)	739 (m)	738 (m)	macroring deformation
748 (vs) +769 (sh)				macroring deformation & C-N-C ring stretching
	770 (m)	773 (m)	772 (m)	C-N stretching & macrocring deformation
	812 (m)	813 (m)	814 (m)	C-N-C macroring breathing
957 (s)	974 (s)	976 (s)	975 (s)	C-N-C macroring breathing
	1076 (m)	1079 (m)	1077 (m)	macroring deformation & C-H bending
1186 (vs)	1203 (vs)	1208 (vs)	1025 (vs)	isoindole in-plane bending
1263 (vs)	1273 (s)	1277 (s)	1275 (s)	isoindole in-plane bending
1315 (s)	1330 (s)	1333(s)	1332 (s)	pyrrole ring stretching
1368 (w)	1382 (m)	1383 (m)	1382 (m)	C-H in-plane benzene bending & pyrrole breathing
1425 (m)	1436 (s)	1441 (s)	1439 (s)	C-C pyrrole stretching & isoindole stretching
1525 (vs)	1525 (vs)	1530 (vs)	1529 (vs)	C-N pyrrole symmetric stretching

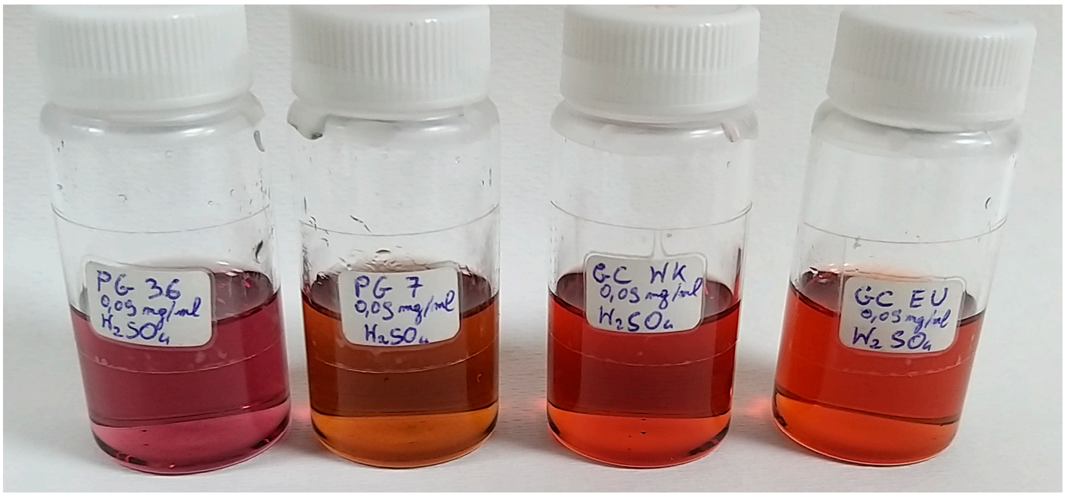

Figure S3 PG36, PG7, AGC and EGC solutions in sulfuric acid, in concentrations 0.09 mg/ml.

Table S2. Mass percentages of elements analyzed by XRF in EGC and AGC and not subjected to RESAP recommended limitations.

**Element**	**EGC**	**AGC**
**Aluminium**	2.56%	2.15%
**Silicon**	0.41%	0.35%
**Phosphorous**	0.22%	0.18%
**Sulphur**	0.02%	0.016%
**Chlorine**	39.2%	32.94%
**Potassium**	0.0053%	0.0044%
**Calcium**	0.075%	0.062%
**Titanium**	0.00001%	0.00001%
**Iron**	0.047%	0.042%
**Gallium**	0.00009%	0.00008%
**Germanium**	0.00001%	0.00001%
**Bromine**	0.039%	0.034%
**Rubidium**	0.00036%	0.00032%
**Strontium**	0.00024%	0.00022%
**Zirconium**	0.027%	0.025%
**Palladium**	0.00005%	0.00006%
**Silver**	0.00046%	0.00051%
**Indium**	0.00008%	0.00016%
**Tin**	0.0028%	0.0043%
**Tellurium**	0.0002%	0.00021%
**Iodine**	0.00023%	0.00001%

**Cesium**	0.00056%	0.00001%
**Lanthanum**	0.00082%	0.00001%
**Cerium**	0.00094%	0.00001%
**Tantalum**	0.00003%	0.00003%
**Tungsten**	0.00042%	0.00037%

Table S3. Correspondence between hazard codes and toxicity. In addition, a colour code was used: **Red=lethality, Orange=Toxicity, Pink=Harmfulness, Bluish= skin related issues, Green=irritation, damage, drowsiness, Bluish in purple field=skin allergic reactions, Bluish in black field=fatal in contact with the skin, Red in black field=carcinogenicity, Red in green field=harm to organs and teratogenicity.** The most common categories we found per hazard are indicated in parenthesis. The lowest the number the higher the risk. Additionally, letters indicate subcategories, A, being associated with the highest risk.

**H301(3)**	**Toxic if swallowed**
**H302(4)**	**Harmful if swallowed**
**H304(1)**	**May be lethal if swallowed or inhaled**
**H310(2)**	**Fatal in contact with skin**
**H311(3)**	**Toxic in contact with skin**
**H312(4)**	**Harmful in contact with skin**
**H314(1C)-Cat.1A**	**Causes severe skin and eye damage**
**H315(2)**	**May cause skin irritation**
**H317(1)**	**May cause an allergic skin reaction**
**H318(1)**	**Causes serious eye damage**
**H319(2A)**	**Causes serious eye irritation**
**H330(2)**	**Fatal if inhaled**
**H331(3)**	**Toxic if inhaled**
**H332(4)**	**Harmful if inhaled**
**H335(3)**	**May irritate the respiratory tract**
**H336(1)**	**May cause drowsiness or dizziness**
**H340 (1B)**	**May cause genetic defects (state route of exposure if it is conclusively proven that no other routes of exposure cause the hazard**
**H350(1A) and (1B)**	**May cause cancer (state route of exposure if it is conclusively proven that no other routes of exposure cause the hazard)**
**H351(2)**	**Suspected of causing cancer (state route of exposure if it is conclusively proven that no other routs of exposure cause the hazard)**
**H360 (1B)- and FD**	**may damage fertility or the unborn child (state specific effect if known) (state route of exposure if it is conclusively proven that no other routes of exposure cause the hazard)**
**H361d (2) and f**	**Suspected of damaging the unborn child**
**H370(1)**	**H370 (1)-Cause damage to organs or state all organs affected, if known state route of exposure if it is conclusively proven that no other routes of exposure cause the hazard.**
**H372(1)**	**Causes damage to organs (or state all organs affected, if known) through prolonged or repeated exposure (state route of exposure if it is conclusively proven that no other routes of exposure cause the hazard)**
**H373 (2)**	**May to cause damage to organs (or state all organs affected, if known) through prolonged or repeated exposure (state route if it is conclusively proven that no other routes of exposure cause the hazard)**

Table S4. Compounds extracted in water and corresponding hazard codes. Chlorinated compounds are reported in **violet**, phthalates in orange and peculiar compounds of AGC in **light blue**. NA = not available, NDAS = non-dangerous according to Sigma. The colours assigned to different hazards indicate: **Red=lethality, Orange=Toxicity, Pink=Harmfulness, Bluish= skin related issues, Green=irritation, damage, drowsiness, Bluish in purple field=skin allergic reactions, Bluish in black field=fatal in contact with the skin, Red in black field=carcinogenicity, Red in green field=harm to organs and teratogenicity**.

RT min	Compound	Hazards	EGC	AGC
**2.18**	Ethyl propanoate	**NA**		⌘
**2.60**	4-methyloctane	**H304(1)**	⌘	
**2.69**	Propyl acetate	**H319(2) H336(3)**	⌘	
**2.70**	2,4-dimethyl-1-heptene	**H304(1)**	⌘	
**2.96**	1,3,5-trimethylcyclohexane	**NA**	⌘	
**3.17**	1,4-dimethylbenzene	**H312(4) H315(2) H332(4)**	⌘	
**3.48**	3,5-dimethyl octane	**NA**	⌘	
**3.85**	Tetramethylsilane	**H302(4) H315(2) H319(2)H335(3)**		⌘
**3.87**	Octamethylcyclotetrasiloxane (D4)	**H361f(2)**	⌘	
**3.94**	4-methyl-2-heptanone	**H315(2) H319(2A) H335(3)**	⌘	
**3.98**	4-ethylheptane	**H304(1)**	⌘	
**4.02**	4-methylnonane	**H304(1)**	⌘	
**4.13**	3-methylnonane	**NA**	⌘	
**4.27**	3-propyl-1-octene	**NA**	⌘	
**4.45**	2-*t*-butyloxytetrahydrofuran	**NA**	⌘	
**4.65**	1-decene	**H332(4)**	⌘	
**4.72**	Di-n-propyl ether	**H336(3)**		⌘
**4.84**	3,3-dimethyloctane	**H318(1)**	⌘	
**4.85**	2-nitropropane	**NA**		⌘
**4.95**	2,6-dimethylnonane	**NA**	⌘	
**4.95**	2-methyl-1-propanol	**H302(4) H332(4) H350(1B)**		⌘
**5.19**	propyl 2-hydroxyacetate	**NA**		⌘
**5.28**	5-methyl-3-hexanol	**H319(2)**	⌘	
**5.50**	3-undecene	**NA**	⌘	
**5.53**	3-methyl-1,2-cyclopentanediol	**H319(2)**	⌘	
**5.75**	4-methyl-2-decene	**NA**	⌘	
**5.94**	Hexadecane	**H304(1)**	⌘	
**6.49**	2-methyl propanoic acid	**H302(4) H312(4)**		⌘
**6.58**	1-hepten-4-ol	**H315(2) H318(1) H335(3)**		⌘
**7.11**	Acetophenone	**H302(4) H319(2)**	⌘	
**7.11**	4,7-dimethylundecane	**NA**	⌘	
**7.16**	Decamethylcyclopentasiloxane (D5)	**H319(2) H331(3)**	⌘	
**7.26**	2,3,6,7-tetramethyloctane	**NA**	⌘	
**7.55**	Methylbenzoate	**H302(4)**	⌘	
**7.61**	4-methyl benzaldehyde	**H302(4) H315(2) H319(4) H335(3)**	⌘	
**7.75**	4,5-dimethyl-4-hexen-3-one	**NA**	⌘	
**8.03**	4-ethyldecane	**NA**	⌘	
**8.07**	1,2,4,5-tetramethylbenzene	**NA**	⌘	
**8.17**	5-methylundecane	**H304(1)**	⌘	
**8.17**	**1-chloroethylbenzene**	**H315(2) H318(1) H334(1) H335(3)**	⌘	
**8.25**	4-methylundecane	**NA**	⌘	

**8.37**	2,3-dimethylundecane	**NA**	✣	
**9.07**	Allyl hexyl oxalate	**NA**		✣
**9.31**	4,8-dimethylundecane	**NA**	✣	
**9.45**	2,3,6-trimethyl-4-octene	**NA**	✣	
**10.13**	1,3-di-*t*-butylbenzene	**NA**	✣	✣
**10.14**	3-methoxy-3-methyl-2-butanone	**H315(2) H319(2A) H335(3)**	✣	
**10.56**	3-hexen-2,5 diol	**H315(2) H319(2)**		✣
**10.64**	2-methyl-1-undecanol	**H317(1)**	✣	✣
**10.86**	1,6-heptadien-4-ol	**NA**		✣
**11.02**	1-nitro pentane	**H315(2) H319(2) H335(3)**		✣
**11.16**	1-nitro hexane	**H315(2) H319(2) H335(3)**		✣
**11.37**	4-methyl benzoic acid	**H302(4) H315(1) H317(1) H319(2)**		
**13.87**	3-methylpenta-1,4-diene-3-ol	**NA**		✣
**14.24**	2,4-di-*t*-butylphenol	**H315(2) H318(1)**	✣	
**14.28**	1,1-diisobutoxy butane	**NA**		✣
**14.83**	2-methyl hexadecane	**NA**		
**15.04**	**Pentachloro benzene**	**H302(4)**	✣	
**15.04**	2,5-di-tert-butyl phenol	**H315(2) H319(2) H335(3)**	✣	
**15.35**	n-heptadecan-1-ol	**H315 H319 H335**	✣	
**15.45**	4-tert-butyl-2,6-diisopropylphenol	**NA**	✣	
**16.25**	2,4,6,8-tetramethyl-1-undecene	**NA**		✣
**16.42**	4,5-dimethyl-1-hexene	**NA**		✣
**16.55**	3-methylpenta-1,4-diene-3-ol	**NA**		✣
**16.77**	10-methyl-4-undecene	**NA**		✣
**16.87**	3-methylpenta-1,4-diene-3-ol	**NA**		✣
**17.76**	n-heptadecyl cyclohexane	**NA**	✣	
**17.86**	3-phenylbuthyl-1-ethylbenzene	**NA**	✣	
**19.49**	hexadecanoic acid	**NA**	✣	
**19.78**	Oleic acid	**H315(2) H319(2) H335(3)**	✣	
**20.91**	2-methyl-3-hexen-2-one	**H302(4) H311(3)**		✣
**20.97**	pentanamide	**H302(4)H315(2)H319(2) H335(3)**		✣
**21.07**	Methyl 16-methylheptadecanoate	**NDAS**	✣	
**21.52**	Octadecanoic acid	**NDAS**	✣	

Table S5. Compounds extracted in acetone and corresponding hazard codes. **violet**, phthalates in orange and peculiar compounds of AGC in **light blue**. NA = not available, NDAS = non-dangerous according to Sigma. The colours assigned to different hazards indicate: **Red=lethality, Orange=Toxicity, Pink=Harmfulness, Bluish= skin related issues, Green=irritation, damage, drowsiness, Bluish in purple field=skin allergic reactions, Bluish in black field=fatal in contact with the skin, Red in black field=carcinogenicity, Red in green field=harm to organs and teratogenicity**, White in a dark red field = compounds were extracted in both acetone and chloroform.

**RT min**	**Compound**	**Hazards**	**EAC**	**AEC**
**2.02**	2-hydroxybutanoic acid	**H315(2) H319(2)**		✣
**2.09**	1, 4-dioxane	**H319(2) H335(3)H351(2)**		✣
**2.21**	4-methyl-3-penten-2-one	**H302(4) H312(4) H332(4)**		✣
**2.37**	Hexamethy Cyclotrisiloxane, (D3)	**H319(2)**	✣	✣
**2.86**	4-hydroxy-4-methyl-2 pentanone	**H319(2)**	✣	✣
**3.37**	Styrene	**H315(2) H319(2) H332(4)**	✣	✣

		H372(1) H361d(2)		
**3.54**	2,4-dimethyl-1-pentene-3-one	H315(2) H319 (2A) H335 (3)		✣
**3.83**	Octamethylcyclotetrasiloxane (D4)	H361f (2)	✣	✣
**4.30**	5-methyl-4-nonene	NA	✣	
**4.30**	5-methyl-3-hexene-2-one	H302(4) H311(3) H332(4)		✣
**4.50**	vinyl hexanoate	H315(2) H319(2)		✣
**4.61**	1,2,3-trimethyl-benzene	H304(1) H315(2) H319(2) H335(3)		✣
**4.62**	Cyclodecane	NDAS	✣	
**4.68**	Decane	H304(1) H315(2) H319(2) H332(4) H335(3)	✣	
**4.70**	2-cyclohexene-1,4-dione	H317 (1)		✣
**4.93**	3,3-dimethyl octane	NA	✣	
**6.07**	2,5-dimethyl-3-hexenyl formate	NA		✣
**6.48**	3,7,10-trimethyl-1-dodecanol	NA	✣	
**7.17**	Decamethylcyclopentasiloxane (D5)	H315(2) H319(2) H331(2)	✣	✣
**7.58**	3,5-dihydroxy toluene	H302(4) H315(2), H319(2) H335(3)		✣
**7.65**	4-methyl benzaldehyde	H302(4) H315(2) H319(2) H335(3)	✣	
**7.83**	2,6-dimethyl-2,5-heptadiene-4-one	NDAS		✣
**8.14**	Levoglucosenone	H302(4) H319(2)		✣
**8.26**	5-methyl-4-undecene	H304(1)	✣	
**8.40**	3,3,5-trimethyl-2-cyclohexen-1-one	H302(4) H312(4) H319(2) H335(3) H351(2)		✣
**8.56**	hexa-3-ene-2-one	NA		✣
**8.83**	Silicic acid	NA		✣
**8.95**	6-dodecene	NA	✣	
**9.01**	3-dodecene	NA	✣	✣
**9.15**	cyclohexa-2-ene-1-one	H301(3) H310(2)H319(2) H330(1) H331(3)		✣
**9.56**	Naphthalene	H302(4) H351 (2)	✣	
**9.65**	Isobutane	H340(1B) H350(1A)		✣
**10.15**	1,3-ditertbutyl benzene	NDAS	✣	
**10.38**	5,6-dihydroxy-4,6,6-trimethyl-2*H*-piran-2-one	NA		✣
**10.45**	Dodecamethylcyclohexasiloxane (D6)	H319(2) H331(3)	✣	✣
**10.67**	7-tetracedene	H304(1)	✣	
**10.88**	1-(2-butenyl)-2,3-dimethyl benzene	NA		✣
**11.30**	2-butyl-1-decene	H304(1)	✣	
**11.57**	1-(1-methylethenyl)-4-(1-methylethyl)-Benzene	NA		✣
**11.67**	1-methylnaphthalene	H302(4)H319(2) H335(3) H336(1) H373(2)	✣	
**11.80**	1,3-dioxolane	H319(2) H360(1B)		✣
**12.05**	3-tetradecene	H304(1)	✣	
**12.35**	4-tetradecene	H304(1) H315(2) H335(3)	✣	
**12.46**	2,5-dimethylfuran	H302(4) H315(2)H317(1) H319(2) H335(3)		✣
**12.92**	Biphenyl	H304(1) H315(2) H319(2) H330(2) H331(3) H335(3)		✣
**13.00**	Tetradecamethylcycloheptasiloxane (D7)	H319(2)	✣	✣
**14.37**	2,4-di-*t*-butylphenol	H315(2) H318(1)	✣	

**14.63**	3-hexadecene	**H304(1)**	⌘	
**14.84**	2-methyl-1-pentadecene	**NA**	⌘	
**15.10**	Hexadecamethylcyclooctasiloxane (D8)	**H319(2)**	⌘	⌘
**15.19**	1-hexadecanol	**H319(2)**	⌘	⌘
**16.04**	15-Crown-5	**H302(4) H315(2) H319(2) H332(4) H335(3)**		⌘
**16.82**	Tetradecamethylheptasiloxane (D9)	**NA**		⌘
**17.18**	2,6-Diisopropylnaphthalene	**H302(4)**	⌘	
**17.89**	1,1'-(3-methyl-1-propene-1,3-diyl)bis-benzene	**NA**	⌘	⌘
**19.17**	pentachloroaniline	**H315(2) H319(2)H320(2B)**	⌘	⌘
**19.27**	12 crown 4	**H330(1)**		⌘
**19.45**	Oleic acid	**H315(2) H319(2) H335(3)**		⌘
**19.70**	hexadecamethyl- Octasiloxane	**NA**		⌘
**19.83**	9-eicosyne	**NA**		⌘
**20.94**	2,6-bis(1,1-dimethylethyl)-1,4-benzenediol	**NA**		⌘
**20.94**	2,6-Di-tert-butylhydroquinone	**NA**		⌘
**21.27**	**4,5,6,7-tetrachloro-1,3-isobenzofuranedione**	**H317(1) H318(1) H334(1) H350(1B) H373(2)**		⌘
**23.02**	**Pentachloro benzamide**	**H302(4) H312(4) H315(2) H319(2) H332(4) H335(3)**	⌘	⌘
**23.60**	**Tetrachloro phthalimide**	**H315(2) H319(2) H335(3)**	⌘	⌘

Table S6. Compounds extracted in CCl_2H_2 and corresponding hazard codes. **violet**, phthalates in **orange** and peculiar compounds of AGC in **light blue**. NA = not available, NDAS = non-dangerous according to Sigma. The colours assigned to different hazards indicate: **Red=lethality, Orange=Toxicity, Pink=Harmfulness, Bluish= skin related issues, Green=irritation, damage, drowsiness, Bluish in purple field=skin allergic reactions, Bluish in black field=fatal in contact with the skin, Red in black field=carcinogenicity, Red in green field=harm to organs and teratogenicity**, White in a dark red field = compounds were extracted in both acetone and chloroform.

**RT min**	**Compound**	**Hazards**	**EGC**	**AGC**
**2.72**	2,4-Dimethyl-1-heptene	**H304(1)**	⌘	
**3.90**	Octamethylcyclotetrasiloxane (D4)	**H361f(2)**	⌘	⌘
**4.75**	Decane*	**H304(1)**	⌘	
**5.00**	2,5,5-trimethylhexane	**NA**		⌘
**5.06**	3,3-dimethyloctane	**H315(2) H319(2) H335(3)**	⌘	
**5.95**	2,4,6 trimethyldecane	**H315(2) H319(2) H335(3)**		⌘
**6.54**	1,4-dimethylcycloctane *	**NA**	⌘	⌘
**6.85**	2,3-dimethyldecane*	**NA**		⌘
**7.69**	3-methylbenzencarbaldehyde	**NDAS**	⌘	
**9.13**	Dodecane	**H304(1) H315(2) H319(2) H335(3)**	⌘	⌘
**9.34**	4-methyldodecane	**H304(1)**	⌘	
**9.69**	4,6-dimethyl dodecane	**NA**	⌘	⌘
**10.34**	2,6,11-trimethyl dodecane	**NA**		⌘
**10.59**	2,7,10-trimethyl dodecane	**NA**		⌘
**10.69**	2- methyl-1-octanol	**NA**	⌘	
**10.83**	2,6,10-trimerthyl dodecane	**H304(1) H315(2) H319(2) H335(3)**	⌘	⌘

**10.94**	2-butyl-1- octanol	**NDAS**	⌘	⌘
**11.78**	5-methyltridecane	**NA**	⌘	
**12.06**	3-methyltridecane	**NA**	⌘	
**12.52**	Tetradecane	**H304(1)**	⌘	⌘
**12.79**	1,3,4,5,6,7-hexahydro-1,1,5,5-tetramethyl-, (2S) -2*H*-2,4a-ethanonaphthalene	**NA**		⌘
**12.89**	biphenyl	**H315(2) H319(2) H335(3)**		⌘
**13.00**	Tetradecamethylcycloheptasiloxane D7	**H319(2)**	⌘	⌘
**13.67**	10-methylnonadecane	**NA**	⌘	
**13.77**	2-isopropyl-6-*t*-butylphenol	**NA**		⌘
**13.94**	Nonadecane	**H304(1)**	⌘	⌘
**14.14**	3,7,11-trimethyl-1-dodecanol	**NA**	⌘	⌘
**14.30**	2,4-di-tert-butyl phenol	**H315(2) H318(1)**	⌘	
**14.47**	n-octadecene	**NA**		⌘
**14.86**	4-ethoxy ethylbenzoate	**H302(4) H315(2) H319(2) H335(3)**		⌘
**15.10**	Hexadecamethylcyclooctasiloxane D8	**H319(2)**	⌘	⌘
**15.38**	9-hexadecene-1-ol	**NA**	⌘	
**15.77**	2,6,10-trimethyl pentadecane	**NA**		⌘
**16.01**	1-Nonadecene	**H304(1)**		⌘
**16.09**	1,3,5-triisopropylbenzene	**NDAS**		⌘
**16.46**	Heptadecane	**H304(1)**	⌘	⌘
**16.64**	2,4-di-*t*-butyl-6-nitrophenol	**NA**	⌘	
**16.76**	2-hexyl-1-octanol	**NDAS**	⌘	⌘
**17.01**	2,6,11,15-tetramethyl hexadecane	**NA**		⌘
**17.23**	2,6-diisopropylnaphthalene	**H312(4)**		⌘
**18.65**	**Dibutyl phthalate**	**H360FD(1B)**		⌘
**18.88**	2,6,10-trimethyl tetradecane	**NA**	⌘	⌘
**19.22**	**Pentachloro aniline**	**H301(3) H311(3) H331(3) H373(2)**	⌘	⌘
**19.26**	7,9-di-tert-butyl-1-oxaspiro [4.5]deca-6,9-dien-2,8-dione	**H315(2) H319(2) H335(3)**		⌘
**19.32**	2-methyl-1-hexadecanol	**NA**	⌘	
**19.96**	2,5-di-tert-Butyl-1,4-benzoquinone	**H315(2) H319(2) H335(3)**		⌘
**20.08**	Bentazone	**H302(4) H319(2)H317(1)**		⌘
**20.17**	4b,5,6,7,8,8a,9,10-octahydro- 4b,8-Dimethyl-2-isopropylphenanthrene	**NA**		⌘
**19.93**	Erucic acid	**H315(2) H319(2) H335(3)**	⌘	
**21.06**	1-Methyl-10,18-bisnorabieta-8,11,13-triene	**NA**		⌘
**21.35**	10-heneicosene	**NA**	⌘	
**21.60**	10-methyl-9-nonadecene	**NA**	⌘	
**21.12**	Heneicosane	**H304(1)**		⌘
**21.43**	10,18-Bisnorabieta-5,7,9(10),11,13-pentaene	**NA**		⌘
**21.64**	Heptacosane	**NA**	⌘	⌘
**21.83**	n-octadecyl acetate	**NA**		⌘
**24.87**	**Diisoctyl phthalate**	**H360FD(1B)**	⌘	
**23.60**	1-Docosene	**H304(1)**		⌘
**25.17**	Octacosane	**NA**		⌘
**27.53**	Tetracontane	**NA**		⌘

**33.58**	Stigmast-5-en-3-ol, oleate*	**NA**		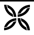
**33.93**	Octadecane	**H304(1) H315(2) H319(2) H335(3)**		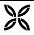

Table S7. Compounds eluted from the chromatographic column with chloroform, and concentrations estimated with a semiquantitative method. **violet**, phthalates in orange.

Compound	EGC Concentration ng/µl	AGC Concentration ng/µl
**Pentachloroethane**	599.03	40.30
**Hexachloroethane**	213.10	229.35
**3,4,6-trichloro-2-methyl phenol**	0.17	0.11
**1,2-dichloro-4-(1-chloroethyl) benzene**	4.04	3.90
Tetradecamethyl cycloheptasiloxane (D7)	0.01	0.01
2,6-di-*t*-butyl-1,4-benzoquinone		0.10
**3-chlorobenzamide**	0.05	
3,3-dimethyl-1-(3H)-isobenzofuranone	0.07	
Hexadecamethyl cyclooctasiloxane (D8)	0.01	0.01
**Pentachlorobenzene**	0.04	0.07
Diethyl phthalate	0.07	0.03
2,3-diphenyl-2-butene		0.40
**Hexachlorobenzene**	0.03	0.24
Butyl tridecyl phthalate	0.35	
**Pentachlorobenzonitrile**		0.26
**4,5-dichloro**phthalimide	0.24	
**Pentachloroaniline**	0.38	3.09
7,9-Di-tert-butyl-1-oxaspiro [4,5]deca-6,9-diene-2,8-dione	0.09	0.16
**Trichlorobenzenamide**	0.06	0.01
**2,3,4,5-tetrachloro aniline**		0.07
Butyl 2-pentyl phthalate	0.16	0.10
**2,3,4,5,6-pentachloro-N-(dichloromethylene)-Benzenamine**		0.03
**Pentachlorophenol**	0.23	0.30
**Tetrachlorobenzamide**	0.19	0.13
**Pentachlorobenzamide**	9.20	8.75
**3,4,5,6-tetrachloro** phthalimide	0.76	1.67
Diisoctyl phthalate	0.06	0.01
